# Impact of Patient-Clinical Team Secure Messaging on Communication Patterns and Patient Experience: Randomized Encouragement Design Trial

**DOI:** 10.2196/22307

**Published:** 2020-11-18

**Authors:** Stephanie L Shimada, Mark S Zocchi, Timothy P Hogan, Stefan G Kertesz, Armando J Rotondi, Jorie M Butler, Sara J Knight, Kathryn DeLaughter, Felicia Kleinberg, Jeff Nicklas, Kim M Nazi, Thomas K Houston

**Affiliations:** 1 Center for Healthcare Organization and Implementation Research (CHOIR) VA Bedford Healthcare System Department of Veterans Affairs Bedford, MA United States; 2 Department of Health Law, Policy, and Management Boston University School of Public Health Boston, MA United States; 3 Division of Health Informatics and Implementation Science Department of Population and Quantitative Health Sciences University of Massachusetts Medical School Worcester, MA United States; 4 Heller School for Social Policy and Management Brandeis University Waltham, MA United States; 5 Department of Population and Data Sciences UT Southwestern Medical Center Dallas, TX United States; 6 Birmingham VA Medical Center Department of Veterans Affairs Birmingham, AL United States; 7 Division of Preventive Medicine University of Alabama at Birmingham School of Medicine Birmingham, AL United States; 8 Center for Health Equity Research and Promotion (CHERP) VA Pittsburgh Healthcare System Department of Veterans Affairs Pittsburgh, PA United States; 9 Center for Behavioral Health, Media and Technology University of Pittsburgh Pittsburgh, PA United States; 10 Mental Illness Research Education and Clinical Center (MIRECC) VA Pittsburgh Healthcare System Department of Veterans Affairs Pittsburgh, PA United States; 11 Innovation, Decision Enhancement & Analytic Sciences (IDEAS) Center George E. Wahlen Veterans Affairs Medical Center Salt Lake City, UT United States; 12 Department of Internal Medicine University of Utah School of Medicine Salt Lake, UT United States; 13 Department of Social and Behavioral Sciences University of California San Francisco, CA United States; 14 KMN Consulting Services, LTD Coxsackie, NY United States; 15 Section on General Internal Medicine Wake Forest School of Medicine Winston-Salem, NC United States

**Keywords:** patient portal adoption, secure messaging, communication, provider autonomy support, patient experience, patient portal, continuous care, patient, design, effectiveness, engagement

## Abstract

**Background:**

Although secure messaging (SM) between patients and clinical team members is a recommended component of continuous care, uptake by patients remains relatively low. We designed a multicomponent Supported Adoption Program (SAP) to increase SM adoption among patients using the Veterans Health Administration (VHA) for primary care.

**Objective:**

Our goals were to (1) conduct a multisite, randomized, encouragement design trial to test the effectiveness of an SAP designed to increase patient engagement with SM through VHA’s online patient portal (My HealtheVet [MHV]) and (2) evaluate the impact of the SAP and patient-level SM adoption on perceived provider autonomy support and communication. Patient-reported barriers to SM adoption were also assessed.

**Methods:**

We randomized 1195 patients at 3 VHA facilities who had MHV portal accounts but had never used SM. Half were randomized to receive the SAP, and half served as controls receiving usual care. The SAP consisted of encouragement to adopt SM via mailed educational materials, proactive SM sent to patients, and telephone-based motivational interviews. We examined differences in SM adoption rates between SAP recipients and controls at 9 months and 21 months. Follow-up telephone surveys were conducted to assess perceived provider autonomy support and self-report of telephone communication with clinical teams.

**Results:**

Patients randomized to the SAP had significantly higher rates of SM adoption than the control group (101/595, 17.0% vs 40/600, 6.7%; *P*<.001). Most adopters in the SAP sent their first message without a motivational interview (71/101, 70.3%). The 10-percentage point difference in adoption persisted a full year after the encouragement ended (23.7%, 142/600 in the SAP group vs 13.5%, 80/595 in the control group, *P*<.001). We obtained follow-up survey data from 49.54% (592/1195) of the participants. SAP participants reported higher perceived provider autonomy support (5.7 vs 5.4, *P*=.007) and less telephone use to communicate with their provider (68.8% vs 76.0%, *P*=.05), compared to patients in the control group. Patient-reported barriers to SM adoption included self-efficacy (eg, not comfortable using a computer, 24%), no perceived need for SM (22%), and difficulties with portal password or login (17%).

**Conclusions:**

The multicomponent SAP was successful in increasing use of SM 10 percentage points above standard care; new SM adopters reported improved perceptions of provider autonomy support and less use of the telephone to communicate with their providers. Still, despite the encouragement and technical assistance provided through the SAP, adoption rates were lower than anticipated, reaching only 24% at 21 months (10% above controls). Common barriers to adoption such as limited perceived need for SM may be more challenging to address and require different interventions than barriers related to patient self-efficacy or technical difficulties.

**Trial Registration:**

ClinicalTrials.gov NCT02665468; https://clinicaltrials.gov/ct2/show/NCT02665468

## Introduction

Secure messaging (SM) is a secure, asynchronous, patient-provider or patient-clinical team electronic communication channel that may help with care coordination and enable more efficient patient-provider interactions [1-3]. Patients use SM to ask questions or to keep their providers and clinical teams informed about their health status in between medical visits [4-6]. Several observational studies and multiple systematic reviews have found that SM can have a positive impact on health outcomes and patient satisfaction for some patients [6-12]. While some providers have expressed concern that SM may be difficult to keep up with and interrupt workflow, SM may help improve productivity by reducing telephone communication and improving visit efficiency [13].

The Centers for Medicare and Medicaid Service Meaningful Use requirement has made SM a common feature of patient portals and tethered personal health records across many health care systems [4,14-16]. In 2004, the Veterans Health Administration (VHA) implemented My Health*e*Vet (MHV), an online patient portal and personal health record that has an SM feature to support communication between patients and their VHA clinical team members [17,18].

However, the majority of VHA patients still do not use SM. As of September 2016, 12% of all VHA patients and 27% of MHV portal users were active users of SM who had sent at least one message to their clinical team in the previous 24 months. At the time of this writing (February 2020), active engagement with SM was at 16.7% of VHA patients. VHA patients tend to be older and face more complex health care needs than the general population and have less socioeconomic means than veterans that do not use the VHA [19]. Previously documented barriers to SM adoption among veterans include lack of awareness about the SM features, not having a need for communication, limited access to technology, low computer literacy, and feelings that their provider does not support SM use [10,20,21]. Facilitators of SM adoption include understanding the purpose for using SM, having a health-related need that aligns with that purpose, and seeing SM as a convenient alternative form of communication [20].

SM has been found to be associated with positive health outcomes, such as improved HbA1c or blood pressure control, and improved antiretroviral adherence and HIV control in several observational studies [7,8,22-25]. However, these studies are prone to selection bias (ie, differential uptake of SM based on health status and other unobserved patient characteristics), and single-site, randomized trials are less generalizable. While research in this field is expanding rapidly, the evidence is not yet mature. Systematic reviews of SM have found many of the studies lacking in rigor [11,12]. To our knowledge, no multisite, randomized trial has tested an intervention to increase SM use or examined its impact on patient-reported outcomes. In practice, randomized trials of SM are difficult to conduct as patients randomized to use SM may never send an SM and control patients may decide to use SM on their own.

To address these challenges, we conducted a multisite, randomized, encouragement trial to test methods to improve patient adoption of SM and to better understand the benefits of SM adoption. Our hypotheses were that patients receiving encouragement to use SM would have higher rates of SM adoption, communicate through telephone less often, and perceive easier access to their provider compared to controls. Through follow-up telephone interviews, we examined barriers to SM adoption to better understand the reasons why patients might be resistant to using SM, despite being encouraged to do so.

## Methods

### Design

As part of a VHA health care operations–funded quality improvement effort, we conducted a multisite, randomized, encouragement design trial, a design that is appropriate for situations when both control and intervention arms have the potential to access or use the treatment being evaluated [26]. Randomized, encouragement trials are a type of trial that retains many of the strengths of randomized clinical trials while allowing patients the opportunity and flexibility to engage in the intervention as they see fit [27]. In a randomized, encouragement trial, participants are randomized to receive encouragement to try the treatment or intervention of interest. It is expected that some participants randomized to receive the encouragement will not try the treatment and that some of the control participants will try the treatment on their own (2-sided noncompliance).

### Setting

Three VHA medical centers participated in the trial. The sites were geographically diverse, located in the western, southern, and northeast regions of the United States, and included a relatively large (~30%) population of rural patients.

### Participants

Veterans were eligible for the trial if they (1) had an authenticated account with the MHV online patient portal, (2) had never used SM, and (3) had a primary care appointment scheduled in the upcoming 2 months. Patients were excluded if they did not have a valid address or telephone number on record.

### Randomization

**Figure 1:**
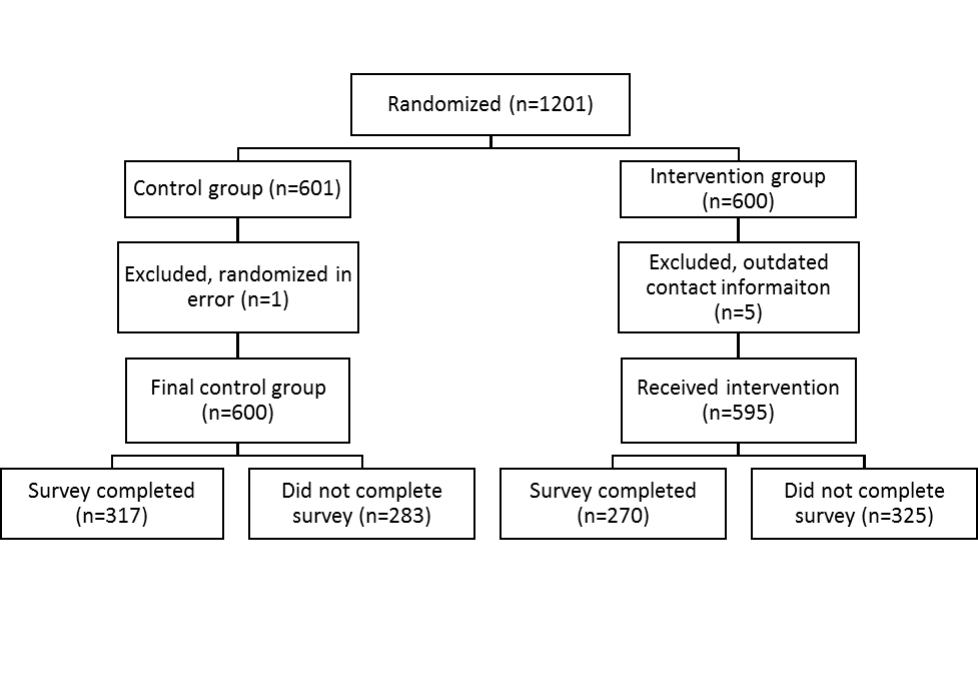
CONSORT diagram.

Each of the 3 sites identified between 500 and 1200 patients who met the eligibility criteria, oversampling patients residing in rural zip codes. All sites used a block randomization table to assign 200 patients to the intervention (encouragement) arm and 200 as controls (Figure 1).

### Intervention

#### Supported Adoption Program

Participants were randomized to be in the Supported Adoption Program (SAP) and receive encouragement to use SM to communicate with their clinical teams or to a control arm in which no encouragement was given. The SAP was comprised of multiple components, including 2 mailings, 2 SMs sent to participants from their primary care team’s SM account, and 1 telephone-based motivational interview. The first mailing was sent in the first week of March 2016, and the last motivational interview was completed in September 2016.

The SAP components were developed to address key constructs of behavioral and motivational theories such as social cognitive theory (expectations, self-efficacy), theory of planned behavior (behavioral intention, subjective norms, attitudes), and the health belief model (perceived benefits, self-efficacy). The components were developed with input from MHV Coordinators, who regularly work with veterans to facilitate patient portal access and SM use. The mailings and team-initiated SMs highlighted reasons why patients like to use SM (such as convenience over telephone communication), how SM can benefit health, and assurance that their clinical teams want to communicate with them through SM. The first mailing contained a letter inviting participants to try SM, a brochure highlighting the advantages of SM, and a mousepad with step-by-step instructions on how to use SM. The first team-initiated SM was sent 3 weeks later and covered similar content as the first mailing. A second mailing was sent 3 weeks later and contained a letter reminding patients of the advantages of SM, a step-by-step instruction sheet for accepting the SM terms and conditions (for those unable to receive the provider-initiated SM), and a magnet with key telephone numbers for local and national support. A second team-initiated SM was sent to patients 2 weeks later with repeated content of the mailings.

Approximately 4 weeks after the second team-initiated SM, project staff at each site checked patients’ portal activity and began making phone calls to patients in the SAP who had not yet sent an SM. The phone calls used motivational interviewing (MI) techniques to elicit behavior change by helping patients explore and resolve ambivalence or barriers to change [28-30]. The project staff were trained via telephone and given scripts for the motivational interviews. Three phone scripts were available based on whether the veteran (1) had read either of the SMs sent, (2) had not read the SMs sent, or (3) could not be sent the SM because they had not yet accepted the SM terms and conditions. Up to 5 attempts were made to reach each veteran. During the MI calls, project staff helped veterans troubleshoot common technical barriers during the call (eg, lost password) as well as other barriers to the use of SM (eg, perceived need, self-efficacy). Veterans were also provided instructions on how to contact their local MHV coordinator and the national MHV helpline.

#### Control Group

Participants randomized to the control group did not receive any component of the SAP.

### Data Collection and Measures

#### Primary Outcome: SM Adoption

The primary outcome measured for this evaluation was SM adoption, which we defined as sending at least one SM during a 9-month period following the initial outreach (March 2016 to December 2016). We continued to monitor SM activity for an additional 13 months (until the end of 2017, when data collection ended) to measure longer-term effects of the intervention. SM activity data were collected via MHV data tables in the VHA corporate data warehouse.

#### Secondary Outcomes

Secondary outcomes were assessed via a telephone survey. The survey administration began approximately 6 months after initiation of the SAP. The survey asked veterans about the use of telephone communication with their health care teams, their perceptions of ease of communication with their health care teams, and perceptions of provider autonomy support, as measured by the Health Care Climate Questionnaire (HCCQ) [31], in the past 6 months. Those who had used SM during the evaluation period were also asked questions about their experience using SM. Patients that completed the survey received a US $20 gift card by mail.

The HCCQ is a series of 15 questions assessing perceived provider autonomy support (eg, “I feel my health care practitioner understands how I see things with respect to my health.”). Patients answer on a scale of 1 (Strongly disagree) to 7 (Strongly agree). The HCCQ score is the average (mean) of these 15 items [31].

To measure the use of a telephone to communicate, patients were asked (yes or no), “In the past 6 months, have you communicated with your health care team by phone?”

To measure ease of communication, patients were asked, “How easy is it for you to communicate with your provider or health care team when you need to?” Patients that responded “easy” or “very easy” were considered to perceive easy access to their providers. Patients who responded, “very difficult,” “difficult,” or “neutral” were considered not to perceive easy access to their providers.

Self-reported barriers to SM adoption were collected during the motivational interviews. These barriers were coded into the following categories: low computer literacy (eg, not comfortable using a computer or navigating to the MHV website), difficulties with access (eg, no computer or internet), login difficulties (eg, lost password or username), effectiveness (eg, did not think they would get a reply), no perceived need for it, preference for in-person or phone communication, privacy concerns, and health-related barriers.

### Secure Message Content

SMs sent by SAP and control patients during the 9-month evaluation period were double-coded for content by 2 research team members who met regularly to discuss and resolve differences in coding. Message content was coded into the following categories: Requests for Information, Requests for Action, and Information Sharing. The Requests for Information (eg, about symptoms, problems, medications, treatments) and Requests for Action (eg, requests for tests, medications, referrals) codes were based on a modified version of the Taxonomy of Requests by Patients [32,33]. The Information Sharing category captured messages that shared information about care obtained from other VA or non-VA providers, informational updates to the team about symptoms, vital readings (eg, blood pressure), or other personal or health-related topics. Previous studies have identified the need to share information with providers as one of the primary motivators of SM use [5,34].

### Analyses

Descriptive statistics and chi-square tests were used to compare characteristics of patients randomized to the encouragement and control groups. We examined the success of the SAP by plotting the cumulative adoption rate over time for each group. Chi-square tests were used to compare adoption rates at 9 months and 21 months. Additionally, each component of the SAP (mailings, proactive SM, motivational interviews) was plotted graphically to examine adoption following each program component. Among patients that sent at least one SM, we used the Wilcoxson rank sum test to test for differences in the number of messages sent in each group. Chi-square tests were used to determine if the coded SM content differed between the groups. For patients that participated in a motivational interview, we identified common barriers to SM adoption and used a chi-square test to estimate the association of each barrier on later adoption. Finally, we compared characteristics of SM adopters to nonadopters regardless of treatment assignment using chi-square tests.

To estimate the effect of SM adoption on patient-reported outcomes, we conducted an intention-to-treat analysis (ITT), analyzing all participants with complete follow-up data as randomized. The ITT analysis compares outcomes in the SAP intervention group to the control group and does not consider whether or not patients sent an SM. ITT analysis is the preferred analytic approach for parallel-arm, randomized trials and more closely resembles real-life practice, where there is noncompliance of treatment. As such, the ITT analysis estimates the “effectiveness” of the SAP intervention rather than the “efficacy” of SM adoption. *t* tests were used for comparisons of continuous outcomes, and chi-square tests were used for comparisons of binary outcomes.

We also conducted a per-protocol analysis. A per-protocol analysis compares patients in control and intervention groups that completed the treatment as originally allocated. (ie, “compliers”) [35]. For the SAP intervention group, this included all patients that sent an SM during the 9-month evaluation period (n=101). For the control group, this included all patients that did *not* send a secure message (n=560). Due to likely differences between compliers in each group, we used multivariable regression analysis, controlling for age, race, gender, marital status, rural residence, and copayment exemption. We used linear regression for continuous outcomes and logit regression for binary outcomes. All analyses were conducted using Stata v.15.1 (Stata Corp, College Station, TX).

## Results

Across the 3 sites, 595 veterans were randomized to receive encouragement, and 600 served as controls. Veteran demographic and socioeconomic characteristics were balanced across the encouragement and control arms (Table 1). Approximately 30% (356/1195, 29.79%) of the veterans resided in rural areas, and 21.59% (258/1195) qualified for copayment exemptions based on their economic means. Approximately 21% (256/1195, 21.42%) of veterans were under the age of 50 years, 9.96% (119/1195) were female, 55.90% (668/1195) were married, and 19.83% (237/1995) were African American.

**Table 1:** Baseline characteristics of patients registered in the patient portal in 2016 who had not yet used secure messaging, with comparisons between the Supported Adoption Program (SAP) and control groups.

Characteristic	Control group (n=600), n (%)	SAP group (n=595), n (%)	Difference, %	χ^2^ statistic	*P* value
African American	112 (18.7)	125 (21.0)	2.3	1.030	.31
Female	61 (10.2)	58 (9.7)	–0.5	0.058	.81
<50 years old	132 (22.0)	124 (20.8)	–1.2	0.239	.62
Married	341 (56.8)	327 (55.0)	–1.8	0.426	.51
Rural residence	172 (28.7)	184 (30.9)	2.2	0.728	.40
Copay exempt	132 (22.0)	126 (21.2)	–0.8	0.120	.73

### Adoption of Secure Messaging at 9 and 21 Months, Compared Between the SAP and Control Groups

**Figure 2:**
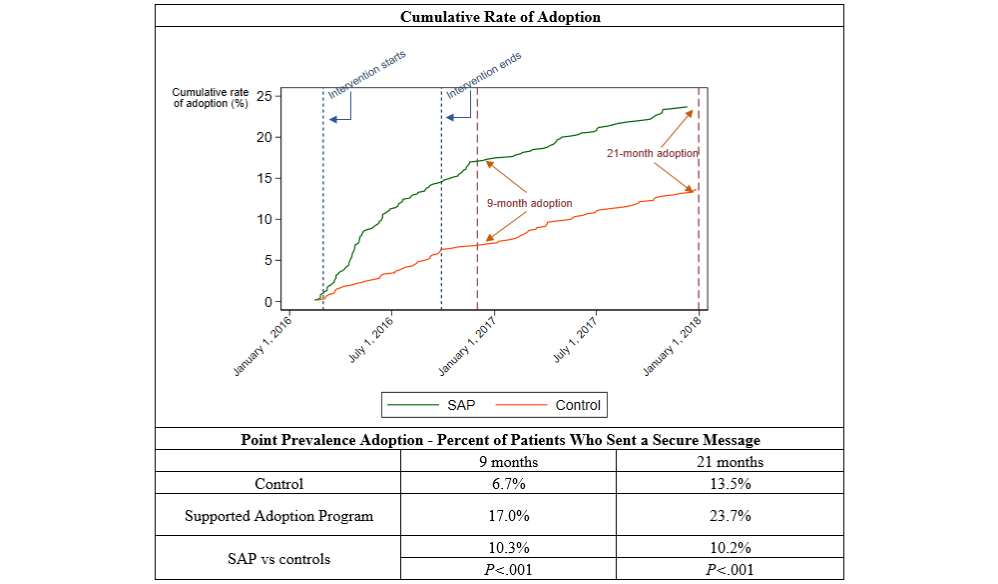
Cumulative rate and point prevalence of secure message adoption among those randomized to the Supported Adoption Program (SAP) and controls.

Veterans that received encouragement were more likely to send an SM than the controls. By the end of 9 months, 17.0% (101/595) of the veterans in the SAP had sent an SM compared to 6.7% (40/600) of the controls (*P*<.001). At 21 months, 23.7% (142/600) of veterans in the SAP had sent an SM compared to 13.5% (80/595) of the controls (*P<*.001; Figure 2).

**Figure 3:**
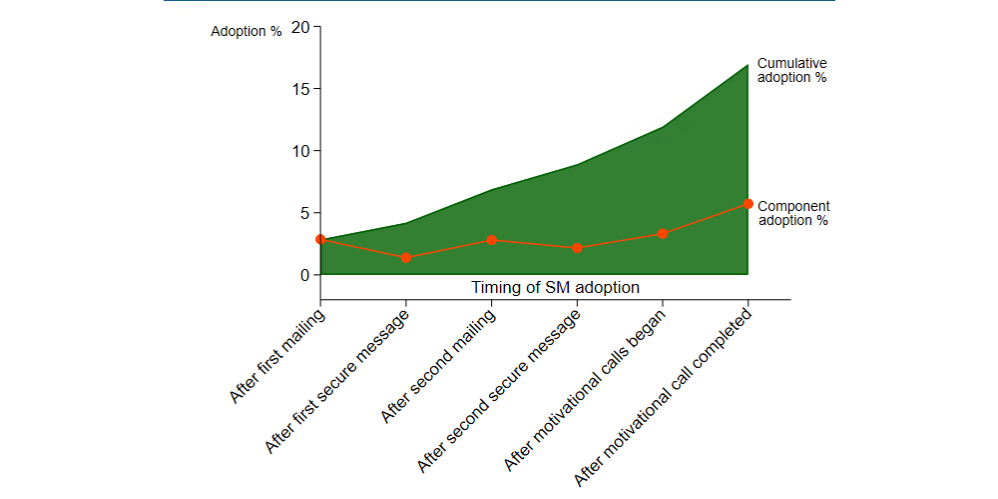
Adoption after each component and cumulative adoption over 9 months of active intervention among patients randomized to the Supported Adoption Program. SM: secure message.

Each component of the SAP (mailings, proactive SM, motivational interviews) was examined separately to explore the contribution of each on the overall rate of adoption (Figure 3). Of the 595 veterans in the encouragement arm who were sent the initial mailing, 17 (2.9%) sent an SM without any additional encouragement. In addition, veterans in this arm were sent 2 proactive encouragement SMs from their primary care team’s SM account and an additional mailing. One-third (198/595) of the veterans could not receive the SMs because they never opted-in to receive secure messages. Of those that received the proactive SMs, 28.0% (111/397) opened and read the messages. This additional encouragement yielded 36 new users for a total of 53/595 (8.9%) new SM adopters before motivational interviews. Of the 542 veterans that were eligible for a motivational interview, 383 interviews were completed, and 30 of those that completed the interview sent an SM (30/383, 7.8%). An additional 18 veterans that could not be reached for the motivational interview (18/159, 11.3%) eventually sent an SM for a total of 101 (101/595, 17.0%) SM adopters at the end of the 9-month intervention.

SM adopters were more likely than nonadopters to be female and younger than 50 years (see Multimedia Appendix 1) We examined the total number of SMs and content of the SMs sent by veterans. A total of 443 new messages were sent during the 9-month evaluation period. Among those who sent an SM, there was no difference between the encouragement and control groups in the average number of messages sent (*P=*.31). SM adopters in both groups sent an average of 2 messages (IQR 1-3). The content of the SMs that were sent by patients was also examined to determine if those in the encouragement arm communicated with their providers differently than the controls. The majority of SMs sent by patients in both groups were information requests or action requests about medications or treatments. We did not find any difference between the 2 groups in the frequency of these types of messages; however, controls were more likely to write to share information about their vitals or provide updates on symptoms or care obtained from other VA or non-VA providers (Multimedia Appendix 2).

During motivational interviews, patients were asked whether they were experiencing any significant barriers to SM adoption. Commonly reported barriers included low self-efficacy (eg, not comfortable using a computer, 24%), no perceived need for SM (22%), and difficulties with portal password or login (17%). The barrier most associated with non-adoption was having portal password or login difficulties (*X*^2^_1_=9.395, *P=*.002). Only 1 patient that identified this as a barrier eventually sent an SM (1/66, 1.5%). Patients that reported at least one significant barrier were much less likely to adopt SM than those that did not identify any (6.5% vs 25.0%, *P<*.001; Multimedia Appendix 3).

### Impact of Secure Messaging on Perceived Health Care Climate and Communication

Table 2 shows the results of the ITT and per-protocol analyses for the 3 follow-up outcomes**.** Compared with the control group, patients that received encouragement were less likely to report using the phone to communicate with their providers (68.8% [SAP group] vs 76.0% [control group], *P=*.05) and were more likely to perceive their provider as autonomy supportive (5.7 [SAP group] vs 5.4 [control group] on a 7-point scale, *P=*.006). Ease of communication did not significantly differ between the two groups. In the per-protocol analysis, SAP recipients that adopted SM were less likely to report using the phone to communicate with their providers compared to the control patients that did not adopt (59.2% vs 77.1%, *P=*.003). Provider autonomy support and ease of communication did not significantly differ between SAP adopters and control nonadopters.

**Table 2:** Impact of the Supported Adoption Program (SAP) and secure message adoption: intention-to-treat (ITT) and per-protocol analyses.

Outcome measure	ITT				Per-protocol			
	SAP (n=270)	Control (n=317)	Difference (95% CI)	*P* value	SAP adopters (n=51)	Control nonadopters (n=293)	Difference (95% CI)	*P* value
Health care climate scale, mean (SD)	5.7 (1.1)	5.4 (1.3)	0.3 (0.1 to 0.5)	.006	5.7 (0.9)	5.4 (1.3)	0.3 (–0.1 to 0.7)	.18
Communicated by phone, n (%)	186 (68.8)	241 (76.0)	–7.2 (–14.5 to 0.0)	.05	30 (59.2)	226 (77.1)	–20.8 (–35.4 to –6.3)	.003
Easy to communicate, n (%)	176 (65.1)	187 (58.9)	6.2 (–1.7 to 14.1)	.12	36 (70.0)	173 (59.0)	11.3 (–2.5 to 25.1)	.13

## Discussion

### Principal Findings

In this randomized trial encouraging SM use, the SAP (encouragement intervention) resulted in an increase in SM use compared with control patients that received usual care. The SAP had a modest but significant impact on overall adoption, with 17% using SM, compared with 7% in the control arm at 9 months, and 24% versus 14% at 21 months.

The majority of new adoption resulted following the low-intensity mailings and SM components. While the motivational interviews resulted in 30 new SM adopters, most new users did not participate in a motivational interview. The mailings or SM components of the SAP would be relatively inexpensive to implement more broadly. The 2 mailings cost approximately US $1.50 to US $5.00 each (depending on whether a small magnet or mousepad was included) and would be scalable to larger groups of patients. We estimate that it would take 5 minutes to send a templated SM with encouragement content to new patient portal users. The motivational interviews took about 30 minutes each to complete and were estimated to cost US $15.50 per completed call based on the average salary of those trained to make the calls. This suggests that, for relatively low effort, health care facilities could engage patients via mailings or SMs highlighting the benefits of SM and addressing barriers to SM use. This type of outreach may also be effective to encourage adoption of other new patient-facing technologies.

In addition to evaluating the program’s effectiveness at getting veterans to use SM, we monitored its impact on self-reported outcomes. Our ITT analysis revealed an increase in perceived provider autonomy support for the encouragement arm compared with the control arm. Increased perceived autonomy support has previously been shown to be associated with improved patient self-efficacy and health management behaviors, and proactive SM from health care teams to patients has been associated with greater perceived autonomy support [36]. It is possible that even templated encouragement to use SM sent from the primary care provider’s team account may be adequate to achieve this benefit. Further, receiving the encouragement resulted in a shifting of patient-clinical team communication modality with a 7% lower rate of self-reported telephone contact in the encouragement arm compared with controls and a 20% lower telephone contact rate among SM adopters in the encouragement arm compared with control nonadopters. Although these are self-reported data, they suggest that SM does not necessarily increase overall communication with providers and may supplement other forms of communication. Future work should evaluate impact on telephone use more objectively.

We analyzed the content of the SM to see whether there were differences in the types of messages sent by those in the encouragement group. Consistent with prior observational studies of SM content, information requests or action requests about medications or treatments were most common [5]. Our content coding did not reveal any significant differences in terms of frequency of requests for information or action; however, control patients were more likely to share information. This may in part be due to relatively small numbers of messages sent and the short timeframe for analysis. Patients may need to become more comfortable with messaging their provider before feeling ready to engage in more complex exchanges. However, it does suggest to us that the encouragement did not cause patients to send meaningless messages simply for the sake of sending an SM and that most patients will wait until they have a clinically relevant reason to SM their clinical teams.

Despite the demonstrated impact of the SAP, the majority of patients still did not engage in SM with their clinical team. We estimated that only one-quarter of those who were sent an SM opened and read the proactive SM. As suggested in the literature, additional encouragement from clinical teams might be needed to increase patient review of SMs [21]. In the VA, veterans are required to set preferences that determine whether they receive email alerts when there is a new SM in their patient portal. Patients who set their patient portal preferences to alert them to new incoming SM are more likely to read them [10]. Veterans who do not get an email alert may not even realize that they have been sent an SM. At the time of this trial, some veterans were unable to receive SMs because they had not accepted the feature’s terms and conditions. This additional step is no longer required as the terms and conditions have been incorporated into those of the portal. Encouragement sent via SM may not be completely effective in systems where patients do not automatically receive message alerts to their regular email accounts or where there are other barriers to receiving messages.

### Limitations

Both encouragement and control arms consisted of existing patient portal registrants. This type of encouragement program may be less effective if targeted to unregistered patients. Further, new users were disproportionately female and under 50 years old, compared with those who did not adopt SM. Thus, additional work may be needed to engage specific subpopulations.

The motivational interviews were conducted over a longer time period due to challenges reaching patients over the phone. As such, some patients that completed calls towards the end of the 9-month evaluation period had less follow-up time than those who completed calls earlier. When we followed all patients for an additional 13 months, SM adoption rates increased overall, but adoption remained consistently higher in the intervention group, as evidenced in Figure 2. Motivational interviews were conducted at each site by different project staff who were jointly trained in MI and used the same telephone scripts. We did not find differences in adoption rates by site, but it is still possible that different project staff may have been more or less effective than others at encouraging participants to start using SM.

While we monitored SM use beyond the 9-month follow-up period, we did not code additional messages sent by SM users after their initial months of use. Additional content coding of messages could have helped to determine whether the SAP may have shaped the content of their messages in any way over time.

### Conclusions

This randomized, encouragement trial demonstrated that an SAP consisting of low levels of outreach to patients to help address known barriers to adoption of SM can successfully increase use. Some patients required more intensive support to begin use; however, our results show that over half of those who began SM use did so without a motivational phone call. About 70% (71/101) of patients that adopted SM did so without a motivational interview, suggesting that more limited outreach without motivational calls would still be effective at increasing SM adoption, while costing substantially less. Receiving information on SM and encouragement to use it had a positive impact on perceived provider autonomy support and, among SM adopters, resulted in lower self-reported use of telephone communication. Importantly, there were no negative impacts on frequency of SM communication or SM content, when compared with control patients who began using SM of their own accord. Low-intensity outreach can successfully engage patients in use of SM, and such use is associated with beneficial secondary outcomes such as improved perceived autonomy support and lower self-reported telephone communication.

## Appendix

Multimedia Appendix 1 Characteristics of secure messaging adopters.

Multimedia Appendix 2 Secure message content.

Multimedia Appendix 3 Barriers to adoption.

## Abbreviations

HCCQ: Health Care Climate Questionnaire
ITT: intention to treat
MHV: My HealtheVet
MI: motivational interviewing
SAP: Supported Adoption Program
SM: secure messaging
VHA: Veterans Health Administration
