# Supplementary material for: Impact of Patient-Clinical Team Secure Messaging on Communication Patterns and Patient Experience: Randomized Encouragement Design Trial
Source: J Med Internet Res. 2020 Nov 18;22(11):e22307. doi: 10.2196/22307 (PMC7710447; doi:10.2196/22307)
Supplement: Multimedia Appendix 1 [file jmir_v22i11e22307_app1.docx]

**Table A. Characteristics of Secure Message Adopters and Non-Adopters**

|  | Non-Adopters (N=1,054) | Adopters (N=141) | Adopters - Non-adopters | χ^2^ | p-value |
| --- | --- | --- | --- | --- | --- |
| African American | 20.1 | 17.7 | -2.4 | 0.444 | 0.505 |
|  |  |  |  |  |  |
| Female | 9.1 | 16.3 | 7.2 | 7.198 | 0.007 |
|  |  |  |  |  |  |
| Under 50 years | 20.1 | 31.2 | 11.1 | 9.089 | 0.003 |
|  |  |  |  |  |  |
| Married | 55.0 | 62.4 | 7.4 | 2.750 | 0.097 |
|  |  |  |  |  |  |
| Rural | 30.1 | 27.7 | -2.6 | 0.347 | 0.556 |
|  |  |  |  |  |  |
| Copayment exempt | 22.3 | 16.3 | -6.0 | 2.631 | 0.105 |
|  |  |  |  |  |  |

Note: Adoption of secure messaging measured at the end of the 9-month study period.
