# Supplementary material for: Impact of Patient-Clinical Team Secure Messaging on Communication Patterns and Patient Experience: Randomized Encouragement Design Trial
Source: J Med Internet Res. 2020 Nov 18;22(11):e22307. doi: 10.2196/22307 (PMC7710447; doi:10.2196/22307)
Supplement: Multimedia Appendix 2 [file jmir_v22i11e22307_app2.docx]

**Table B. Content of Secure Messages Sent by Controls and SAP Recipients**

|  | % Control Messages (N=108) | % SAP Messages (N=336) | SAP - Controls | χ^2^ | p-value |
| --- | --- | --- | --- | --- | --- |
| Requests for information | 29.6 | 36.0 | 6.4 | 1.47 | 0.225 |
|  |  |  |  |  |  |
| Requests for action | 44.4 | 46.7 | 2.3 | 0.17 | 0.679 |
|  |  |  |  |  |  |
| Information sharing | 21.3 | 3.9 | -17.4 | 33.32 | <0.001 |
|  |  |  |  |  |  |
