# Supplementary material for: Impact of Patient-Clinical Team Secure Messaging on Communication Patterns and Patient Experience: Randomized Encouragement Design Trial
Source: J Med Internet Res. 2020 Nov 18;22(11):e22307. doi: 10.2196/22307 (PMC7710447; doi:10.2196/22307)
Supplement: Multimedia Appendix 3 [file jmir_v22i11e22307_app3.docx]

**Table C. Self-Reported Barriers to Adoption Among SAP Recipients**

|  | Reported as Barrier, N (%) | Reported as a barrier and later adopted, % | Did not report as a barrier and later adopted, % | χ^2^ | p-value |
| --- | --- | --- | --- | --- | --- |
| Low self-efficacy | 91 (23.8) | 7.7 | 14.8 | 3.058 | 0.080 |
|  |  |  |  |  |  |
| No/limited effectiveness | 18 (4.7) | 0.0 | 13.7 | 2.845 | 0.092 |
|  |  |  |  |  |  |
| Difficulties with access | 55 (14.4) | 5.5 | 14.4 | 3.292 | 0.070 |
|  |  |  |  |  |  |
| No perceived need | 84 (22) | 7.1 | 14.8 | 3.347 | 0.067 |
|  |  |  |  |  |  |
| Prefer phone or in-person | 6 (1.6) | 0.0 | 13.3 | 0.918 | 0.338 |
|  |  |  |  |  |  |
| Privacy/security concerns | 8 (2.1) | 0.0 | 13.4 | 1.231 | 0.267 |
|  |  |  |  |  |  |
| Login difficulties | 66 (17.3) | 1.5 | 15.5 | 9.395 | 0.002 |
|  |  |  |  |  |  |
| Health limitations | 4 (1.0) | 0.0 | 13.2 | 0.609 | 0.435 |
|  |  |  |  |  |  |
| Any barrier | 245 (64.1) | 6.5 | 24.8 | 25.830 | 0.000 |
|  |  |  |  |  |  |

Note: Barriers were self-reported during motivational interviews for SAP recipients that had not yet sent a secure message. Patients followed between 64 and 198 days after interview depending on timing of motivational interview (mean = 155 days, SD = 29 days).

.
